# The Development and Validation of a Nomogram for Predicting Cancer-Specific Survival and a Risk Stratification System for Patients with Primary Gastrointestinal Melanoma

**DOI:** 10.5152/tjg.2023.22711

**Published:** 2023-08-01

**Authors:** Shaotian Qiu, Rui Chen, Zhenjun Yu, Shuai Shao, Haixia Yuan, Tao Han

**Affiliations:** 1 Nankai University Faculty of Medicine, Tianjin, China; 2 Department of Gastroenterology and Hepatology, Tianjin Union Medical Center of Tianjin Medical University, Tianjin, China; 3 Department of Hepatology and Gastroenterology, The Third Central Clinical College of Tianjin Medical University, Tianjin, China

**Keywords:** Mucosal melanoma, gastrointestinal tract, prognostic nomogram, risk stratification, cancer-specific survival

## Abstract

**Background/Aims:**

The aim of our study was to develop and validate a nomogram to predict cancer-specific survival and make a risk stratification system for primary gastrointestinal melanoma.

**Materials and Methods:**

Patients with primary gastrointestinal melanoma in the Surveillance, Epidemiology, and End Results database between 2000 and 2018 were included and randomly divided into the training and validation cohort (8:2). A prediction nomogram of cancer-specific survival was constructed based on the risk factors identified in the multivariate Cox regression. Calibration curve, time-dependent receiver operating characteristic, and decision curve analysis were performed. Further, a risk stratification system was developed based on the nomogram.

**Results:**

A total of 433 patients were included. The nomogram was constructed based on age, site, and tumor size, Surveillance, Epidemiology, and End Results (SEER) stage, and therapy. The area under the curves of the nomogram predicting 6-, 12-, and 18-month cancer-specific survival were 0.789, 0.757, and 0.726 for the internal validation and 0.796, 0.763, and 0.795 for the external validation. Calibration curves and decision curve analysis were performed. Further, patients were divided into 2 risk subgroups. The Kaplan–Meier analysis and the log-rank test showed that the risk stratification made well differentiation of patients with different risks of cancer-specific survival.

**Conclusion:**

We developed and validated a practical prediction model of cancer-specific survival and a risk stratification system for patients with primary gastrointestinal melanoma, which might be available in clinical practices.

**Main Points**

- Patients with primary gastrointestinal melanoma (PGIM) had poor prognosis with an 18.0-month median cancer-specific survival (CSS) time.
- Age, site, tumor size, Surveillance, Epidemiology, and End Results (SEER) stage, and therapy are independent risk factors associated with CSS in PGIM.
- A prediction nomogram for the CSS of PGIM patients was constructed.
- The risk stratification built in this study made well differentiation of patients with different risks of CSS.

## Introduction

Primary mucosal melanoma (MM) is an aggressive cancer arising from the uncontrolled proliferation of melanocytes located in the mucosal membrane. Compared to cutaneous melanoma, primary MM is far rarer but carries a poorer prognosis. Bishop and Olszewski^1^ found that the 5-year survival was 34% in MM while it was 89% in cutaneous melanoma. Primary gastrointestinal melanoma (PGIM), as an even rare subtype of MM, has the worst prognosis among MM.^2^ Previous studies reported that the median survival of PGIM was 12.0-19.5 months.^3,4^ Meanwhile, the incidence of PGIM was found to be on the rise.^5^ Even though, the rareness of the tumor has consequently made it difficult to conduct sufficient research in a single center with a large population and there is little evidence regarding the risk factors of cancer-specific survival (CSS) in PGIM.

The Surveillance, Epidemiology, and End Results (SEER) database contains information about the incidence and prognosis of various cancers and has become a good way to investigate rare tumors such as PGIM. Studies of PGIM based on the SEER database including the epidemiology, therapy, and prognosis have been published.^4,6-12^ However, neither the prediction model of CSS nor the risk stratification system of PGIM patients was constructed. A reliable prediction model for CSS and risk-stratification system of PGIM patients may help clinicians to differentiate high-risk patients from low-risk patients, and thus make a more favorable decision. Considering the short median survival time of PGIM, we believed that the analysis focusing on short-term cancer-specific mortality may be more necessary. Hence, the aim of our study was to establish and validate a practical nomogram for predicting the 6-, 12-, and 18-month CSS, as well as to develop a risk-stratification system for PGIM patients.

## Materials and Methods

### Patient Enrollment and Data Collection

All data of patients with PGIM were retrieved and collected from the SEER database, 18 Registries (with plus data, 2000-2018, Nov 2020 Sub; http://seer.cancer.gov/). The inclusion criteria were as follows: (i) primary sites located in the gastrointestinal (GI) tract (identified by International Classification of Diseases for Oncology-Morphology codes, C15.0 to C21.8 and C26.0 to C26.9); (ii) ICD-O-3 codes for the histological type were melanoma (8720-8780); (iii) ICD-O-3 histological behavior as malignant; (iv) the label of “primary by international rules” as primary to identify the primary melanoma; (v) complete clinical information. The exclusion criteria were as follows: (i) patients without confirmed positive histology or exfoliative cytology; (ii) patients with missing/unknown cause of death; (iii) patients with incomplete follow-up data. Only the first lesion of PGIM was included for analysis in patients with multiple lesions of PGIM.

Data of baseline information, tumor characteristics, therapy provided, and follow-up data were collected. Baseline information included age (20-40 years, 41-64 years, >64 years), gender (female, male), marital status [married, others (unmarried, widowed, single, etc.)], race (White, Black, and others), previous cancer history (no, yes). Tumor characteristics included site [upper GI tract (esophagus, stomach), intestine, anorectum], tumor size (≤3 cm, 3-5 cm, ≥5 cm), and SEER stage (localized, regional, distant). A localized cancer is defined as a malignancy limited to the site of origin. A regional cancer is defined as direct tumor extension beyond the limits of the site of origin and/or regional lymph nodes involved. Distant tumors are tumor cells that have broken away from the primary tumor, have travelled to other parts of the body, and have begun to grow at a new location. Therapy provided was grouped into 4 groups: none, surgery only, radiation/chemotherapy only, and surgery combined with radiation/chemotherapy. Follow-up data included CSS and survival time (month). The unit of follow-up time was month. For patients with more than zero days of survival but did not reach 1 month, the survival time was recorded as 0.5 month to differentiate them from those who had zero day of survival.

As the SEER database is available to the public, approval from a local ethics committee is not necessary.

### Statistical Analysis

All of the cases were randomly divided into either the training or validation cohort (the split ratio was 8:2). The training cohort was used to establish the prediction model and to construct the nomogram and risk stratification system, while the validation cohort was used to validate the model.

Univariate and multivariate Cox regressions were performed to identify the significant variables. Those variables identified were applied to establish the nomogram to predict CSS. To evaluate the predictive accuracy of nomogram, the C-index and the calibration plots were performed (bootstraps with 300 resample). Time-dependent receiver operating characteristic curves and the area under the curve (AUC) were used to evaluate the discriminative power of the nomogram. In addition, decision curve analysis (DCA) for nomogram was also performed. Furtherly, patients were divided into different risk-stratification groups based on the cut-off value of the total point calculated by the nomogram. Survival analysis between 2 groups was conducted using the Kaplan–Meier method and log-rank test. All the statistical analyses were performed by R software version 4.1.3. The cut-off value of the total point calculated by the nomogram was identified by X-tile (Version 3.6.1). A 2-sided *P* < .05 was considered statistical significant.

## Results

### Patient Characteristics and Prognosis Outcome

**Figure 1.**
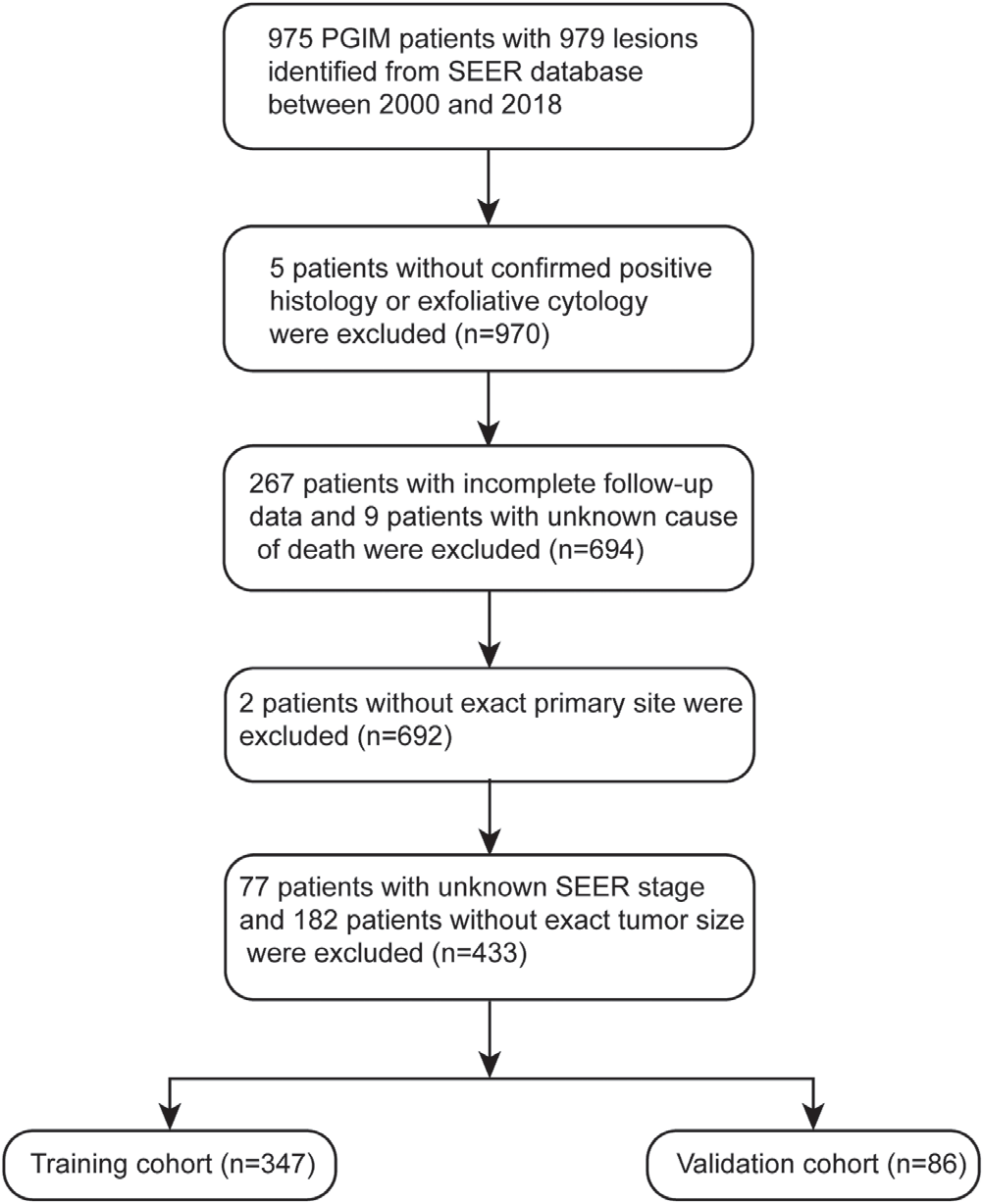
The inclusion and exclusion flowchart of patients with primary gastrointestinal melanoma.

A total of 433 PGIM who met the inclusion criteria were included (Figure 1). The demographic and clinical characteristics of patients were shown in Table 1. Most PGIM patients (63.5%) were older than 64 years old. Of the patients, 43.0% (n = 186) were male while 57.0% (n = 247) were female. Tumors were more commonly located in the anorectum (86.6%) than the upper GI tract (5.3%) and intestine (8.1%). The proportion of tumor size ≤3 cm, 3-5 cm, and ≥5 cm were 43.0%, 27.9%, and 29.1%, respectively. Of the patients, 34.4%, 34.2%, and 31.4% were at localized, regional, and distant stages. As high as 8.5% of patients did not accept any therapy, while 58.7% accepted surgery only, 9.2% accepted radiation/chemotherapy, and 23.6% accepted surgery combined with radiation/chemotherapy.

For all patients, the median CSS time was 18.0 months (95% CI: 14.7-21.3). The 6-, 12-, and 18-month CSS rates were 77.6%, 59.6%, and 48.6%. The median CSS time was longer in tumors located in the intestine and anorectum compared to those located in the upper GI tract (anorectum vs. upper GI tract: 18.0 months vs. 9.0 months, *P *= .035; intestine vs. upper GI tract: 22.0 months vs. 9.0 months, *P *= .075). The median survival months were 33.0 months for the localized stage, 21.0 months for the regional stage, and 7.0 months for the distant stage (*P *< .001). Compared to patients who accepted any therapies, patients without treatment had a poorer prognosis (no therapy vs. therapy: 5.0 months vs. 19.0 months, *P *< .001).

All of the cases were randomly divided into either the training (n = 347, 80%) or validation cohort (n = 86, 20%). The median CSS was 18.0 months (95% CI: 14.5-21.5) and 18.0 months (95% CI: 10.7-25.3) in the training and validation cohorts, respectively (log-rank test, *P* = .241). The baseline characteristics were balanced between the training and validation cohorts (Table 1).

### The Establishment and Validation of Nomogram for Cancer-Specific Survival

Data from the training cohort was included to identify the significant variables. In the univariate Cox regression of CSS, age, site, tumor size, SEER stage, and therapy were identified as significant (*P *< .05). Multivariate Cox regression showed that age, site, tumor size, SEER stage, and therapy were independent risk factors for CSS and were applied to the establishment of nomogram (*P *< .05, Table 2).

**Figure 2.**
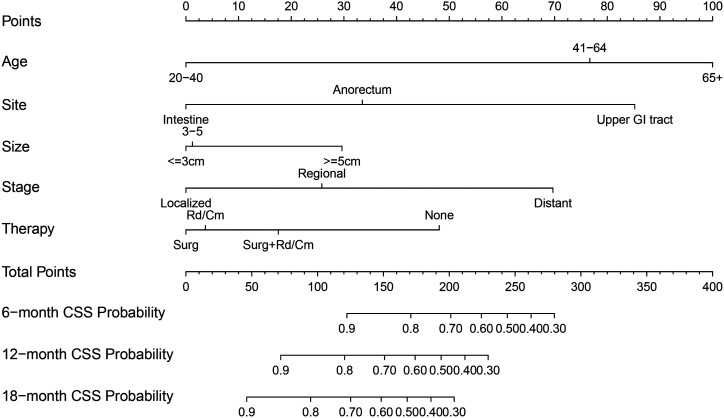
Nomogram for predicting 6-, 12-, and 18-month cancer-specific survival of primary gastrointestinal melanoma patients.

**Figure 3.**
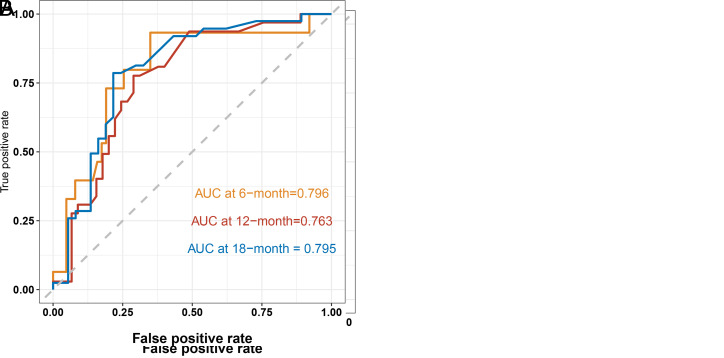
Time-dependent receiver operating characteristic curve for predicting patients’ 6-, 12-, and 18-month CSS of nomogram in the (A) training cohort and (B) validation cohort.

**Figure 4.**
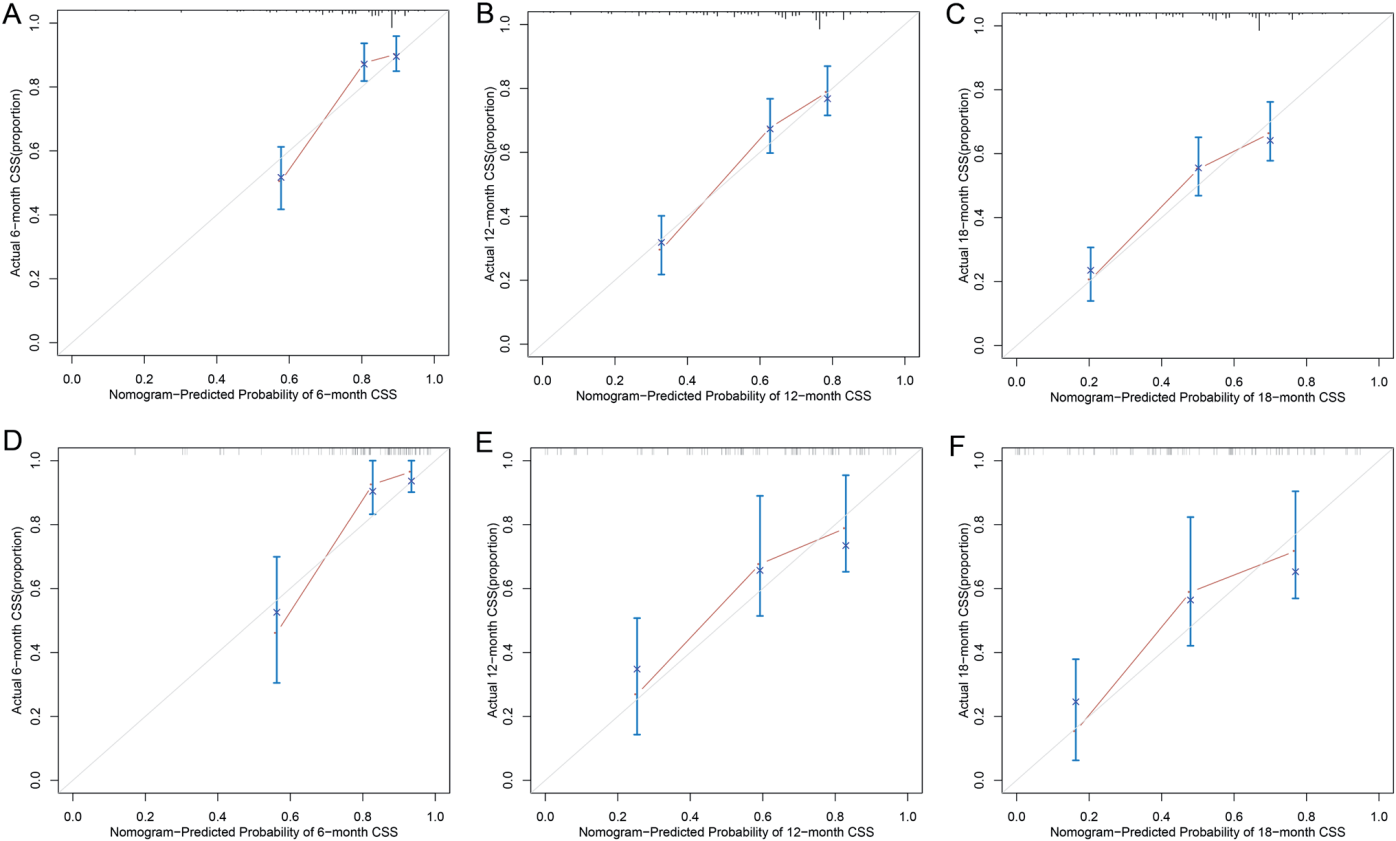
Calibration curve for 6-, 12-, and 18-month cancer-specific survival in the (A-C) training cohort and (D-F) validation cohort.

**Figure 5.**
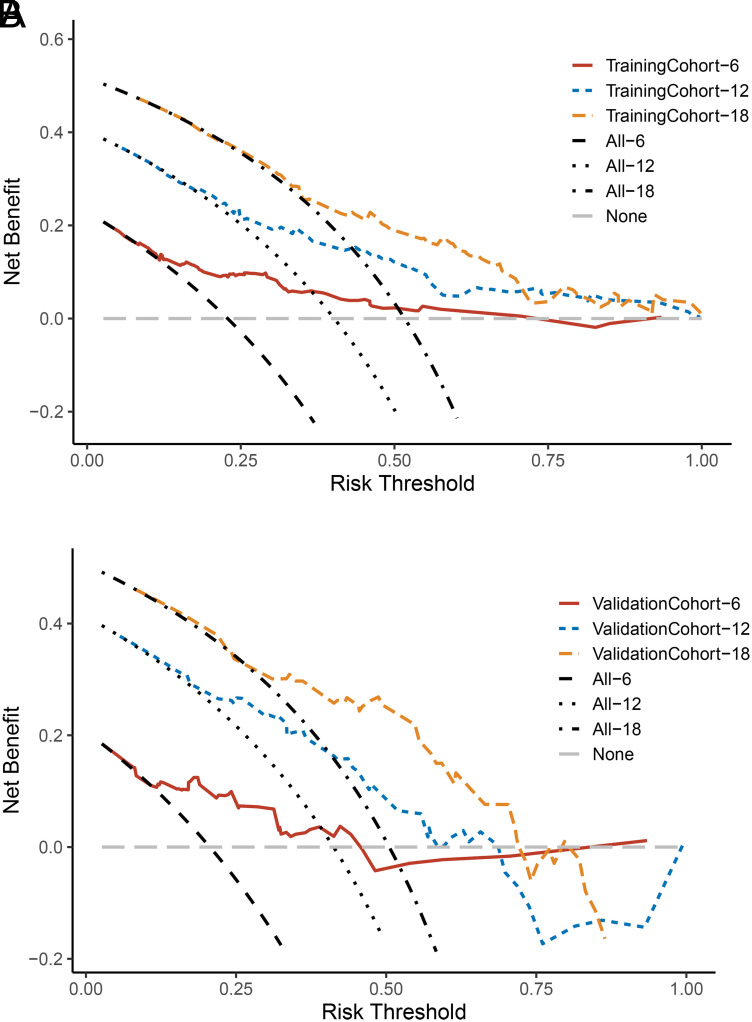
The decision curve analysis for predicting 6-, 12-, and 18-month cancer-specific survival in the (A) training cohort and (B) validation cohort.

The nomogram was virtually displayed for predicting 6-, 12-, and 18-month CSS (Figure 2). The point of different levels in all variables is shown in Table 3. Internal validation and external validation were performed. The AUCs of the nomogram predicting 6-, 12-, and 18-month CSS were 0.789, 0.757, and 0.726 for the internal validation (Figure 3A) and 0.796, 0.763, and 0.795 for the external validation (Figure 3B). The calibration curves for CSS at 6-, 12-, and 18-month showed good consistency between the actual observation and the nomogram prediction in the internal validation (Figure 4A–C) and external validation (Figure 4D–F). The DCA of 6-, 12-, and 18-month CSS demonstrated good net benefits across a range of risk thresholds both in the internal validation and external validation (Figure 5A and 5B).

### Risk Stratification System Based on the Nomogram Model

Based on the cutoff value of the total points calculated by the nomogram, a risk stratification system was developed. Patients were divided into 2 groups according to the point: low-risk (point: 0-182) and high-risk (point: 183-333). The Kaplan–Meier analysis was performed to compare the CSS of different risk stratification groups.

**Figure 6.**
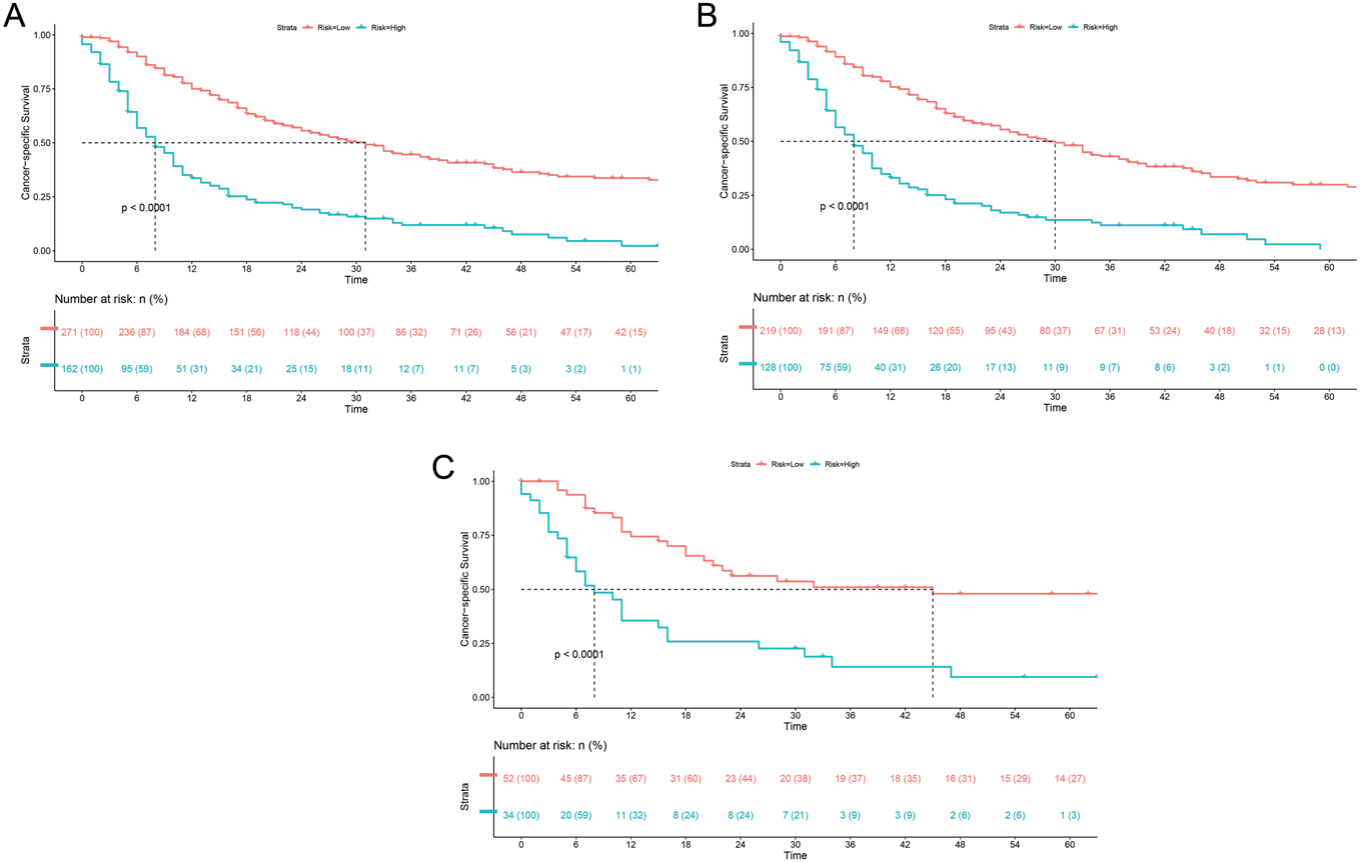
The Kaplan–Meier survival curve of risk stratification groups for cancer-specific survival in (A) whole patients, (B) the training cohort, and (C) the validation cohort.

In all patients, the 6-, 12-, and 18-month CSS were 90.0%, 75.1%, and 63.4% in the low-risk subgroup and 56.9%, 33.7%, and 23.8% in the high-risk subgroup. The median CSS was 31.0 months (95% CI: 24.5-37.5) in the low-risk subgroup and 8.0 months (95% CI: 6.2-9.8) in the high-risk subgroup (Figure 6A). In the training cohort, the 6-, 12-, and 18-month CSS were 89.2%, 75.2%, and 63.0% in the low-risk subgroup and 56.5%, 33.1%, and 23.2% in the high-risk subgroup. The median CSS was 30.0 months (95% CI: 23.8-36.2) in the low-risk subgroup and 8.0 months (95% CI: 5.8-10.2) in the high-risk subgroup (Figure 6B). In the validation cohort, the 6-, 12-, and 18-month CSS were 93.8%, 74.4%, and 65.5% in the low-risk subgroup and 58.2%, 35.6%, and 25.9% in the high-risk subgroup. The median CSS was 45.0 months (95% CI: 0.0-112.8) in the low-risk subgroup and 8.0 months (95% CI: 4.2-11.8) in the high-risk subgroup (Figure 6C). The Kaplan–Meier and log-rank test showed that the risk stratification made well differentiation of patients with different risk of cancer-specific mortality.

## Discussion

Mucosal melanoma is rarely observed in the GI tract. Nowadays, the reports of PGIM were mainly case reports, case series, or retrospective studies based on a small population.^13-15^ A large population-based study in a single center is difficult to carry out due to the rare nature of the tumor. Studies of PGIM with a relatively large population published today were mainly based on the SEER database. In 2021, Badakhshi et al^10^ developed a nomogram for predicting the overall survival of PGIM patients. However, the prediction model for CSS and risk-stratification system for PGIM patients have not been established yet. In this study, through the investigation of the risk factors of CSS, we built the prognostic model for CSS of patients with PGIM and stratified the patients into low- and high-risk subgroups. We believed this model would be available in clinical practices including prognosis evaluation, individual treatment decisions, and management of patients.

The PGIM most commonly occurs in the sixth and seventh decades of life.^4,16^ In this study, 63.5% of patients were older than 65 years old, and the older patients had a poorer prognosis compared to the younger. The PGIM can arise in every part of the GI tract. The most common site is the anus (31.4%), followed by the rectum (22.2%), while PGIM located in the esophagus, stomach, and intestine are quite rare.^4^ The prognosis of PGIM differs in different sites. The median survival time of different GI tract reported was 12 months for the esophagus, 5 months for the stomach, 16 months for the small intestine, and 14-20 months for the anorectum.^4,17-19^ In this study, PGIM located in the upper GI tract had a worst prognosis compared to those located in the intestine and anorectum, which was in accordance with previously reported. Previous study about anorectum melanoma revealed that patients with smaller tumor size had better survival outcome.^20^ Our study also found that tumor size ≥5 cm was associated with a 1.58-fold cancer-specific mortality compared to those ≤3 cm, which implicated that tumor size is a prognostic factor of CSS in PGIM patients.

The stage of tumor has been proved to be associated with the prognosis of various tumors. The most widely used staging system is the American Joint Committee on Cancer (AJCC) TNM staging system; however, it has not been applicated to PGIM. A previous study by Kahl et al^12^ found that the median CSS time of anorectal differed at different SEER stages (localized: 33.0 months, regional: 18.0 months, distant: 6.0 months), which implicated the value of SEER stage in PGIM. Hence, the SEER stage system was used in this study. The multivariate Cox regression revealed that the SEER stage showed a good predictive value of CSS in PGIM patients in this study. A higher SEER stage was associated with a poorer prognosis.

Only 34.4% of patients in this study were diagnosed at an early stage and 65.6% were diagnosed at an advanced stage. The low survival rate may be related to the late discovery of melanoma lesions as well as the lack of standardized treatments.^21^ The rarity nature of the tumor has led to a paucity in the treatment protocol. Surgical resection is considered the optimal therapy of PGIM and studies have identified that surgery significantly increased the survival prognosis.^4,22^ The controversy mainly exists in the choice of surgical procedure. For anorectal melanoma, early research found that extensive surgery led to better disease control,^23^ while recent studies concluded that the extensive surgery led to better local control but did not improve survival prognosis compared to conservative surgery.^8,11^ This might be because the “early tumor spread to distant sites thus overcoming the potential benefits of local control.”^8^ In view of the deficient evidence of the benefit of extensive surgery, as well as the related morbidity of the procedure, some researchers advocated conservative treatments as the first-line therapy.^11,24,25^ For PGIM in other sites, studies comparing extensive surgery and conservative surgery were absent due to the limited cases. In this study, the results of multivariate Cox regression demonstrated that surgery is a protective factor of CSS, but the effect of different surgical options was not investigated and further studies are warranted.

Primary gastrointestinal melanoma patients with advanced stage may be poor surgical candidates. Adjuvant therapies including radiation, chemotherapy, and immunotherapy may therefore represent promising treatment choices for them. At present, the effect of adjuvant therapy remains unknown. Studies in other MM revealed that the radiation may improve local control but did not affect survival.^26,27^ Khaliq et al^28^ used 4 cycles of chemotherapy in a gastric melanoma patient and achieved a resolution of lesion, while another study reported a poor prognosis of an intestinal melanoma patient who accepted chemotherapy.^29^ Immunotherapy, which was considered a promising way to treat cutaneous melanoma, was not that effective in PGIM. Bolzacchini et al^30^ reported a case of gastric melanoma treated with BRAF inhibitor vemurafenib, followed by monoclonal antibody direct against CTLA-4 ipilimumab. The patient passed away 11 months after being diagnosed. Nevertheless, the studies published now were all case reports or case series and thus not sufficient to make meaningful conclusions. Further research is warranted to identify the effect of adjuvant therapy in PGIM.

Here, we developed a prognostic prediction model for 6-, 12-, and 18-month CSS based on the factors we identified in the multivariate Cox regression. The AUC were all above 0.7 in the training cohort and validation cohort, which indicated a good predictive ability of the model. The calibration curve revealed good consistency between the predicted and actual probabilities of survival. Besides, DCA showed the good net benefits of the prediction model across a range of risk thresholds, which reflect the clinical value of the model. Further, we stratified the patients into 2 risk subgroups based on the total points calculated from the prediction model (Table 3 and Figure 6). The Kaplan–Meier curve revealed that the high-risk group had a worse prognosis compared to the low-risk group. The model successfully differentiated the patients that were at a high risk of mortality from those not and may be available in the clinical management of PGIM patients.

This study still encountered some limitations. First, this is a retrospective SEER database-based study, and only the patients with complete follow-up data were included in this study, which might induce a selection bias. Second, the detailed course of radiation and chemotherapy were not available, meanwhile, data regarding the immunotherapy data were not included in the database, which might be associated with the prognosis. Third, although we developed a prediction model and validated it internally and externally, further external validation with data from multi-center is not available due to the rarity of the neoplasm. Nevertheless, considering the rarity of PGIM, our study still has a practical function in the predictive of CSS prognosis and risk-stratification of PGIM.

In conclusion, we investigated the factors associated with the CSS of PGIM and developed a practical prediction model for CSS. Patients were further divided into different risk subgroups. The model may be available in clinical practice and help clinicians in predicting prognosis and individualizing the treatment for PGIM patients with different risks.

## Figures and Tables

**Table 1.** Baseline Information and Clinical Characteristics of the Included Patients

Variables	Total (n = 433)	Training Cohort (n = 347)	Validation Cohort (n = 86)	*P*
Age, years, n (%)				
20-40	13 (3.0%)	9 (2.6%)	4 (4.7%)	.604
41-64	145 (33.5%)	117 (33.7%)	28 (32.6%)	
>64	275 (63.5%)	221 (63.7%)	54 (62.8%)	
Gender, n (%)				
Male	186 (43.0%)	153 (44.1%)	33 (38.4%)	.395
Female	247 (57.0%)	194 (55.9%)	53 (61.6%)	
Marital status, n (%)				
Others	195 (45.0%)	160 (46.1%)	35 (40.7%)	.398
Married	238 (55.0%)	187 (53.9%)	51 (59.3%)	
Race, n (%)				
White	371 (85.7%)	298 (85.9%)	73 (84.9%)	.137
Black	24 (5.5%)	22 (6.3%)	2 (2.3%)	
Others	38 (8.8%)	27 (7.8%)	11 (12.8%)	
Prior cancer, n (%)				
No	338 (78.1%)	271 (78.1%)	67 (77.9%)	1.000
Yes	95 (21.9%)	76 (21.9%)	19 (22.1%)	
Site, n (%)				
Upper GI tract	23 (5.3%)	17 (4.9%)	6 (7%)	.469
Intestine	35 (8.1%)	26 (7.5%)	9 (10.5%)	
Anorectum	375 (86.6%)	304 (87.6%)	71 (82.6%)	
Tumor size, n (%)				
≤3 cm	186 (43.0%)	156 (45%)	30 (34.9%)	.132
3-5 cm	121 (27.9%)	97 (28%)	24 (27.9%)	
≥5 cm	126 (29.1%)	94 (27.1%)	32 (37.2%)	
SEER stage, n (%)				
Localized	149 (34.4%)	121 (34.9%)	28 (32.6%)	.655
Regional	148 (34.2%)	115 (33.1%)	33 (38.4%)	
Distant	136 (31.4%)	111 (32%)	25 (29.1%)	
Therapy, n (%)				
None	37 (8.5%)	29 (8.4%)	8 (9.3%)	.904
Surg	254 (58.7%)	203 (58.5%)	51 (59.3%)	
Rd/Cm	40 (9.2%)	31 (8.9%)	9 (10.5%)	
Surg + Rd/Cm	102 (23.6%)	84 (24.2%)	18 (20.9%)	

Cm, chemotherapy; GI, gastrointestinal; Rd, radiation; SEER, the Surveillance, Epidemiology, and End Results database; Surg, surgery.

**Table 2.** Univariate and Multivariate Cox Regression of CSS

Variable	Univariate Cox regression		Multivariate Cox regression	
	HR (95%CI)	*P*	HR (95%CI)	*P*
Age, years				
20-40	1 (Ref)		1 (Ref)	
41-64	2.01 (0.92-9.23)	.069	3.27 (1.02-10.47)	.046
>64	3.70 (1.18-11.63)	.025	4.69 (1.47-15.02)	.009
Gender				
Male	1 (Ref)			
Female	1.18 (0.91-1.53)	.203		
Marital status				
Married	0.83 (0.64-1.07)	.156		
Others	1 (Ref)			
Race				
White	1 (Ref)			
Black	1.07 (0.64-1.79)	.786		
Others	1.11 (0.71-1.72)	.652		
Prior cancer				
No	1 (Ref)			
Yes	0.95 (0.69-1.31)	.746		
Site				
Upper GI tract	1 (Ref)		1 (Ref)	
Intestine	0.38 (0.18-0.82)	.014	0.27 (0.12-0.60)	.002
Anorectum	0.44 (0.25-0.78)	.005	0.45 (0.25-0.81)	.008
Tumor size				
≤3 cm	1 (Ref)		1 (Ref)	
3-5 cm	1.27 (0.92-1.75)	.142	1.02 (0.73-1.42)	.911
≥5 cm	1.98 (1.47-2.66)	<.001	1.58 (1.13-2.21)	.008
SEER stage				
Localized	1 (Ref)		1 (Ref)	
Regional	1.49 (1.08-2.06)	.015	1.49 (1.07-2.07)	.017
Distant	3.05 (2.21-4.20)	<.001	2.94 (2.04-4.23)	<.001
Therapy				
None	1 (Ref)		1 (Ref)	.005
Surg	0.24 (0.15-0.38)	<.001	0.48 (0.28-0.79)	.029
Rd/Cm	0.48 (0.27-0.86)	.014	0.50 (0.27-0.93)	.086
Surg + Rd/Cm	0.30 (0.18-0.49)	<.001	0.62 (0.36-1.07)	.005

Cm, chemotherapy; GI, gastrointestinal; Rd, radiation; SEER, the Surveillance, Epidemiology, and End Results database; Surg, surgery.

**Table 3.** Points Assigned for Variables

Variables	Point
Age, years	
20-40	0
41-64	77
>64	100
Site	
Upper GI tract	85
Intestine	0
Anorectum	33
Tumor size	
≤3 cm	0
3-5 cm	1
≥5 cm	30
SEER stage	
Localized	0
Regional	26
Distant	70
Therapy	
None	48
Surg	0
Rd/Cm	4
Surg + Rd/Cm	18

Cm, chemotherapy; GI, gastrointestinal; Rd, radiation; SEER, the Surveillance, Epidemiology, and End Results database.
